# The revolution next door

**DOI:** 10.1111/1468-4446.13131

**Published:** 2024-07-08

**Authors:** David Calnitsky, Kaitlin Pauline Wannamaker

**Affiliations:** ^1^ Department of Sociology University of Western Ontario London Ontario Canada; ^2^ Department of Sociology McGill University Montréal Québec Canada

**Keywords:** democracy, inequality, reform, revolution

## Abstract

This paper explores the cascading influence of revolutionary moments on democracy and inequality, not at home, but across borders. We use data on revolutions and other social upheavals over the past 120 years and examine their cross‐national impact on a range of variables in neighboring countries. Engaging with debates on whether substantial democracy and equality increases require extraordinary circumstances, our research investigates whether revolutionary activities induce consequential spillovers, such as policy concessions from elites in neighboring contexts. In exploring spillover effects, the paper examines how significant events in one nation influence social life in adjacent ones. It encompasses an analysis of 171 countries over two centuries, connecting data on revolution with democracy and equality metrics, and hypothesizing that elite fear of revolutionary contagion may necessitate democracy and equality concessions to mitigate potential uprisings. Findings suggest neighboring revolutions positively impact domestic democracy and equality levels. We observe significant increases in an index of democracy and two indices of economic egalitarianism, although one of the egalitarianism measures is robust to all model specifications. Additionally, we find that isolated “protest‐led ousters” can moderately increase suffrage and one of our indices of egalitarianism, while coups do not seem to impact democracy or inequality variables. By examining various upheaval types and outcomes across time and space, the study illuminates the causal relationship between global mobilizations and local changes, providing insights into how global events inform domestic outcomes.

## 1 INTRODUCTION

Revolutionary change has been a defining feature of the modern world. The sparks of revolution can be ignited by war, crises, and widespread discontent, and the consequences of such upheaval are often far‐reaching (Acemoglu & Robinson, 2012; Goldstone, 2014; Goodwin, 2005; Kuran, 1989; McAdam et al., 1996; Skocpol, 1979; Tilly, 1978). But how far can they reach? Surely, they can impact a country's developmental trajectory: they are very clearly impactful across time. There is no understanding French, Chinese, or Russian history without reaching back in time to come to grips with the lineage of revolution. But do they reach across space? Can revolutionary enthusiasm spread across borders to impact democracy and equality *beyond* its point of origin? Revolutionaries focused their attention on the impact of the revolution at home, but they also debated its role abroad, asking whether revolution could beget revolution (see for example, Carr, 1978; Mandel, 1978). An adjacent question asks not about the spread of revolution, but about the role of revolution in fomenting reform. Instead of inquiring into the consequences of revolution at home, this paper asks about its cascading influence on inequality and democracy beyond national borders. Revolution may or may not generate positive domestic consequences, but if the turmoil instills enough anxiety in neighboring elites, they may grant concessions that would be otherwise rebuffed. Can revolutionary spillovers mutate into reformist ends?

Putting this question in its broadest form, one can inquire into the origins of the welfare state and democracy. Do these outcomes emerge out of ordinary or extraordinary circumstances? Are they the products of politics as usual or are they birthed from rare external shocks? Thomas Piketty (2014) has suggested that the Russian Revolution may have motivated elites in Europe to make the policy concessions that created the welfare state. The historian Walter Scheidel (2017) identified transformative revolution as one of the “four horsemen” of leveling; he contends that violent social upheavals, such as modern revolution, have been crucial in generating significant declines in inequality. Scheidel suggests that inequality is so deeply entrenched a social problem it cannot be substantially addressed through gradual reform, but rather requires more drastic and violent means to bring about lasting change. Others have argued that a perceived revolutionary threat can bring about the expansion of suffrage and economic redistribution at home (Acemoglu & Robinson, 2006; Aidt & Jensen, 2014; Paster, 2013; Przeworski, 2009). On the other hand, research by Omar Wasow (2020) suggests that violence is more likely to bring about backlash than change. But the mechanisms Wasow references are domestic; perhaps the backlash effect is less likely to play out in a cross‐national context—a neighbor may observe the romance but not the repercussions of revolution. The stakes of the debate are high because if substantial increases in democracy and equality require unpredictable external events, then social change may be largely outside our control. While this paper does not cast doubt on reform, it may be the case that revolutionary moments are unique in their broad social consequences.

More recently, Rasmussen and Knutsen have tried to test some of these claims in the context of the Russian Revolution (2022). They seek to operationalize elite perceptions of revolutionary threat in Europe and use a measure of whether representatives from various countries traveled to Moscow to participate in the first Comintern meeting in 1919. Participation in these meetings may have signaled the credibility of internal revolutionary threats to domestic elites in other European countries. Their cross‐country analysis and case study on Norway illustrate the profound influence such external threats can exert on domestic policy decisions, often resulting in policy concessions from the ruling elites. They find that states facing greater threats expanded social transfers and reduced work hours after 1917 to a larger extent than did other countries. Our paper asks a similar question, but is pitched on a global scale, considering all revolutionary moments as well as coups and protest‐led ousters since 1800 and their impacts on a range of inequality and democracy measures in all neighboring contexts.^1^

To generalize this hypothesis, we look at the universe of revolutionary mobilizations and consider the effects in neighboring countries. Revolution itself may or may not generate beneficial outcomes directly—and this paper does not examine the experiences in Russia, China, Cambodia, Iran, or anywhere else—but perhaps there are positive effects from the *potential* of one, transmitted by way of a turbulent upheaval in a nearby country.

Do the neighbors of revolutionary states increase welfare policies out of fear in the subsequent years? A given country may see increases in democracy and equality in the years following a revolution in a neighboring state. Fear of spillover may bring concessions. Or, to argue the contrary, perhaps anxious elites respond to portending contagion with domestic repression and retrenchment. If Wasow's (2020) mechanisms hold across borders, violent mobilizations might generate retrenchment because violence helps elites to frame the political discourse. In either case, revolutionary activity may have important unintended consequences in non‐revolutionary contexts.

Perhaps the most important debate inside twentieth century communism took on a very similar question, where Stalin and Trotsky were the main antagonists. Is “socialism in one country” possible? Or must revolutionary socialism spread around the world—a view that was called “permanent revolution” in the lingo of the time—to be successful? Stalin argued the former and Trotsky the latter; Trotsky lost the debate with an ice pick in his head.

The controversy can be reframed in the less violent and more academic language of spillover effects (Brock & Durlauf, 2001; Calnitsky & Latner, 2017; Durlauf, 2001; Glaeser et al., 1996; Sah, 1991; Scheinkman, 2008). How much influence do major events in one country have on social life in adjacent ones? Studying social change in one country may require studying it in other ones.

This paper is global in scope, exploring the relationship between revolution in one country and inequality and democracy in nearby countries. Our analysis includes 171 countries over two centuries, merging data on equality and democracy with data on revolution (Coppedge et al., 2021; Miller, 2021a, 2021b). We examine whether countries that are exposed to revolution in a neighboring nation within the prior 5 years are more likely to see a rise in equality and democracy as a consequence. The hypothesized mechanism runs through elite fears of revolutionary contagion (Huntington, 1968; Przeworski, 2009). Could the birthpangs of a nearby revolution incubate one at home? Elites may feel it necessary to placate these brewing social upheavals with a cocktail of democracy and equality.

This research represents a novel contribution to the field, as the first study to examine our question on a global scale over an extended period of time. Previous research has typically focused on specific case studies or individual revolutions, rather than considering the broader global trends and patterns (Rasmussen & Knutsen, 2024). Our study builds upon the work of Knutsen and Rasmussen, who explore the impact of the Russian Revolution on policy change in a forthcoming book. While they focus on policy changes, we consider inequality and democracy. Perhaps more importantly, they focus on a single revolution, where our paper takes a broader approach, examining quantitative data on all of the revolutionary moments over the past two centuries and their regional effects. The Russia case is plausible, but other examples might work in the same way. An awareness of the importance of democratic governance and the ills of inequality in Central America might have been downstream from revolution in El Salvador and Nicaragua in 1979. More directly, reforms in 2011 in Morocco might have been, in some part, downstream from a revolutionary moment in Egypt. Indeed, while Morocco did not itself experience the kinds of upheavals witnessed in Egypt, Tunisia, or Libya, its democracy scores in the Varieties of Democracy data tick upwards when we compare before and after 2010 (for details, see Miller et al., 2012). The revolutions of 1848 (Hobsbawm, 1996; Sperber, 2005) present another interesting case. A number of countries that did not experience revolutions directly—namely, Denmark, Norway, The Netherlands, and arguably Russia—emerged from the moment with increases in their democracy score. We find that revolutions next door usually means good news for domestic levels of democracy and equality. We observe significant increases in an index of democracy and two indices of egalitarianism, although some of these effects are not robust to all model specifications. Moreover, we look not only at revolutions per se, but also similar forms of social upheavals, namely coups and “protest‐led ousters,” led respectively by military elites and grassroots social movements rather than organized rebels. In doing so, we can expand our scope to include not only time and space, but also a variety of mobilization tactics. We find that isolated protest‐led ousters can moderately increase suffrage and one of our indices of egalitarianism, while coups do not seem to move the needle much on democracy or inequality. We present a nuanced picture of how global shake‐ups can spark local changes. By taking a holistic view across time and space, and considering a range of upheaval types and a range of outcomes, we are able to shed new light on the causal sequence from mobilization to social change, and better understand how global events shape domestic outcomes.

## 2 THERE GOES THE NEIGHBORHOOD

The relationship between revolution and inequality has been a topic of longstanding interest within the social sciences, with a wealth of research examining how revolutions can shape the distribution of wealth and resources within societies. Early work in this area, such as that by Goldstone (2014) and Tilly (1978), focused on the broader socio‐political context of revolution and its consequences for societies undergoing radical change. More recent research has sought to quantify the impact of revolution on a range of specific outcomes connected to social welfare.

One of the key findings in this literature is that revolutions can have a powerful impact on inequality within national borders (Acemoglu & Robinson, 2012). However, the direction and magnitude of this impact can vary significantly depending on the specific context and outcomes of the revolution. Some studies have suggested that revolutions may lead to increased inequality in the short term, as elites seek to protect their wealth and power in the face of instability and uncertainty (Huntington, 1968). Others have argued that revolutions may lead to more equal distribution of resources in the long term, as they create opportunities for redistributive policies and more inclusive political systems (McAdam et al., 1996).

The concept of “spillover effects,” or the idea that events or policies in one country can have unintended consequences for their neighbors, has a long history in the social sciences. Typically, the concept is applied to individuals where the treatment effect on Person A can shape outcomes for that person and those changing outcomes can also impact Person B nearby (see Sampson et al., 2002). Przeworski (2009) underscores the far‐reaching impact of revolutionary threats in neighboring countries by noting that pressures from the marginalized or impoverished segments of the population can spur positive policy changes, like franchise extension and economic redistribution. If a program targeted at one young adult leads them to stay in school, that behavioral change might also impact a friend who then decides to do the same. The same indirect effects have been studied in the context of labor market participation (Calnitsky & Latner, 2017), crime (Glaeser et al., 1996; Sah, 1991), health‐outcomes (Sampson et al., 2002), poverty (Veit‐Wilson, 1987), and education (Solon et al., 2000) and a range of other outcomes. Early work applied to a cross‐national context focused on the economic impacts of trade and financial flows between countries (Krugman, 1991). For example, trade linkages can transmit economic shocks or policy changes from one country to another, as changes in the demand for a country's exports or the availability of imported inputs can affect its economic performance (Baldwin et al., 1999). Similarly, financial flows can transmit shocks and changes in economic policy between countries, as movements in capital can affect the availability of credit and the exchange rate (Eichengreen & Hausmann, 1999). Finally, migration can transmit economic and social changes between countries, as movements of people can affect labor markets and the distribution of skills and resources within societies (Hatton & Williamson, 1998). Scholars have begun to examine the potential spillover effects between countries in a range of areas, other forces such as innovation (Acemoglu, Robinson, and Verdier, 2017), state spending (Baicker, 2005), democratization (Starr, 2021), and as we have noted in the case of Russia, revolution (Rasmussen & Knutsen, 2022). How much influence do major events in one country have on social life in adjacent ones?

Overall, the literature suggests that revolution can have complex and multifaceted effects in the short and long run. While some studies have found that revolution may lead to increased inequality in the short term, others have argued that it may create opportunities for more equal distribution of resources in the long term (Hatton & Williamson, 1998; Huntington, 1968; McAdam et al., 1996). When a revolution happens in a given country so many changes transpire at once it is hard to isolate the causes of change. But when it happens in an external context it may be more straightforwardly interpreted as a unified external shock and causal pathways might be more easily traced. The concept of spillover effects offers a useful framework for understanding the ways in which global events and processes can shape domestic outcomes, and this is what we draw out in this paper.

## 3 HYPOTHESES

This study focuses on investigating the effect of revolution and other social upheavals on our democracy and inequality outcomes in neighboring countries. In line with the literature on spillover effects, we hypothesize that:

H 1 If a neighbor has a revolution, nearby countries will experience positive unintended consequences in the form of strengthened democracy and reduced inequality.

Again, the proposed mechanism is that elites favor concessions when they fear the alternative is a complete loss of social power. If revolutionary events not only topple a government, but see former leaders jailed or killed, those leaders have strong incentives to reform. In the name of self‐preservation, leaders may endorse downward transfers of income and power. The counter‐hypothesis suggests that repression works better than concession, and in case of revolutionary anxiety, elites may crack‐down to avoid revolution at home. Here, we should expect either that democracy and equality move in the opposite direction, or that they remain stable.

We also consider transformative events that closely resemble revolutions but differ on the “who's” and the “why's.” As noted above, two prominent varieties of political upheaval are *coups* and *protest‐led ousters*. While *revolution* is defined here as a turnover in state power by rebel forces, *coups* are turnovers initiated by elites such as military actors. In contrast, *protest‐led ousters* are grassroots turnovers from below, excluding rebels (Miller, 2021b).

We argue that the above‐mentioned political upheavals each have unique socioeconomic implications. In particular, we propose that each upheaval type—revolution, coup, and protest‐led outster—is likely to have different impacts on neighboring elites. The events differ and will be interpreted differently by neighbors; as such, the strategies deployed in response by nearby elites will also differ. Democracy and equality may plausibly diffuse some kinds of social crises but not others. For instance, a revolution in a neighboring country might impact ordinary people. If regional conditions are somewhat similar in terms of discontentment, despair, or frustration, a successful example of social change might generate a copycat effect. Democracy and equality might work in this case as an appeasement strategy. However, a coup in a neighboring country might not resonate the same way, insofar as coups are primarily rooted in elite discontent. This elite‐based power struggle often has limited implications for ordinary citizens; competing military elites might seek little mass support. As such, the appeal of democracy and equality as a tool to mollify the masses may have little efficacy. They are not the tools to best address elite conflict. Moreover, neighboring political leaders might perceive coups as more context‐specific and less detrimental to their regime: they might attribute nearby coups to the unique dynamics of elite squabbles in their separate contexts. The more an event is considered a result of distinctive traits, the less likely a spillover effect becomes. Without a perceived threat, leaders will feel no urgency to change their policy or decision‐making approach, thereby maintaining the status quo. Alternatively, if there are regional military alliances, the spillover effect could be additional regional coups on the one hand, or “coup‐proofing” on the other (Powell, 2012). This might make for anti‐democratic consequences among neighbors. For these reasons, we propose that:

H 2a If a neighbor has a coup, nearby countries will not experience strengthened democracy and reduced inequality.

The counter‐hypothesis suggests that regional coups are connected to one another. Perhaps regional military alliances make spillover effects plausible. And perhaps coups are uniquely well organized, and therefore more threatening, than are non‐elite led turnovers of power. This might mean that it is important to get the mass of the population on board with current leadership, in order to stave off any threats to the regime. In this case, democracy and equality are not designed to placate an unhappy population, but are used to strengthen a network of support for the current leadership. Indeed, there is evidence that domestic coups can generate local democratization (Miller, 2021b; Powell & Thyne, 2011; Thyne & Powell, 2016); perhaps it is true for neighbors too.

Finally, we can also consider grassroots turnovers from below. A push from below may more directly require the appeasement of mass discontent, from the perspective of neighboring heads of state. Elites may believe that a discontented population will be contented with some measure of equality and democracy. If so, neighboring countries may attempt to mitigate this real or perceived threat by offering policy concessions designed to curtail political opposition or mobilization. Tarrow's concept of “modular collective action” (Tarrow, 2022) might be applicable to understanding the dynamics of protest‐led ousters. The theory posits that successful protest tactics and organizational strategies can be adopted and adapted by movements in neighboring countries, effectively creating a replicable “module,” that might foster a wave of similar grassroots movements. This is consistent with attempts to model protest “contagion” (Beger et al., 2014) and the idea of tipping points that produce “cascades of protest” (Kuran, 1991). And if the threat is real, it could prompt neighboring states to implement peremptory reforms. On the other hand, isolated protest‐led ousters may be poorly organized and therefore less threatening. Domestic repression may work best in the context of contagion from poorly organized grassroots rebellions. Moreover, relative to revolution—when rebels are likely to be better organized and more dangerous—elites may be more likely to exit unscathed, thereby lowering their costs of inaction. Therefore, we hypothesize that:

H 2b If a neighbor experiences an isolated protest‐led ouster, nearby countries will experience moderate increases in democracy and declines in inequality.

The counter‐hypothesis in this case would be the same as the revolutionary case. Overall, we predict protest‐led ousters without other shocks to yield results similar to those of revolutions, albeit in a weaker form. Whatever the direct impact of a political upheaval, the impact may be positive for those witnessing it from a distance.

## 4 DATA AND METHODS

### 4.1 Data

The data for this study comes from two main sources: the Varieties of Democracy (V‐Dem) database (Coppedge et al., 2021) and the Archigos dataset as adapted by Michael Miller for his book *Shock to the System* (2021b).

Combined, these two sources allow us to analyze the impact of revolutionary neighbors on equality and democracy at home. The V‐Dem database is a widely used source of information on democracy and political institutions. It contains over 350 million data points on nearly 200 countries, covering a wide range of indicators related to democratic governance and political behavior.

The Archigos dataset is a comprehensive collection of information on revolutionary moments as well as other social upheavals from 1875 to 2015 (Goemans et al., 2016). The data was collected from primary sources, including government documents, news articles, and interviews with key informants. Miller (2021a) updated the data and coded in new observations extending backwards in time, capturing social upheaval from 1800 to 2013. After merging these sources, we have a final dataset including our dependent variables on equality and democracy, our independent variables on revolution and other transformative political events, and our covariates. Depending on the observations present in the dependent variable, our regression analysis begins either in 1800 or 1900 and includes 171 countries.

Our study uses six dependent variables from V‐Dem to assess the influence of transformative mobilizations on democracy and inequality. Including different measures representative of democracy and inequality allows us to capture the defining characteristics of these overarching concepts. Percent democracy, constructed by Boix et al. (2013), is a binary measure that quantifies the extent of democratic attributes within a country, including the functioning of the government, political participation, civil liberties, and the fairness of elections (Coppedge et al., 2021). In addition, the electoral democracy index is an ordinal measure, designed to reflect the quality and breadth of democratic processes that are specifically linked to elections (Alexander et al., 2012). Suffrage, another democracy measure, pertains to the universal right to vote in political elections, capturing a fundamental pillar of democratic societies. The study also incorporates the measure of legal equality, which could be interpreted as an equality or a democracy variable. It assesses the degree to which laws within a society ensure equal treatment of individuals and offer protection against discrimination. The egalitarian component index, a composite measure, encapsulates the extent of egalitarian values within society, covering aspects of economic equality, social equality, and gender equality. Lastly, the variable of equal distribution captures the degree to which a country's resources are distributed evenly among its citizens, serving as a key indicator of socioeconomic equity. The equal distribution variable is one of the three components of the egalitarian component index; for this reason, it is perhaps the most narrowly material measure of equality in our data.

To examine how democracy and inequality outcomes may vary across different forms of mobilization, we use three independent variables. These variables capture global data spanning from 1800 to 2013, encompassing 16,869 country‐years. Our data on revolution defines the concept, as noted above, as a rebel‐led government turnover, not a mere “revolutionary moment,” as it is defined, for example, in the Cross‐National Time Series data archive (Banks & Wilson, 2017). The data records 93 total instances and includes familiar cases such as the Chinese, Cuban, Iranian, and Nicaraguan Revolutions where leaders were removed by rebels. We use this variable to test our main hypothesis (H1). The Archigos measure of rebel‐led turnover is the primary source, and it is supplemented by additional instances from Nardulli et al., 2013, as well as Geddes et al., 2018. Instances of rebel victory in civil war aligning with irregular turnovers utilized data from Archigos, and hand‐coded cases from 1800 to 1814 were also incorporated by Miller (2021a).

Our second independent variable, coups, correspond to coercive regime changes carried out by government elites, examples of which include Iran in 1953, Greece in 1973, and Brazil in 1964. This independent variable aligns with our secondary hypothesis (H2a) positing that regional coups do not lead to positive democracy and inequality outcomes in neighboring countries. Data from 1950 to 2013 was constructed using Archigos, Powell and Thyne (2011), and Marshall and Marshall (2018). Pre‐1950, the data primarily uses Archigos data and is supplemented with the PIPE Przeworski, 2013 and CNTS Banks & Wilson, 2017 databases and hand‐coded cases by Miller (2021a), resulting in a total of 412 coup years.

The third and final independent variable we include are protest‐led ousters. We posit that such events in a neighboring country could trigger modest improvements in democracy and inequality outcomes in surrounding states (H2b). The Archigos dataset, which catalogs government turnovers instigated from below excluding rebel‐led instances, is supplemented with data on instances of regime failures stemming from popular protests (Geddes et al., 2018; Nardulli et al., 2013). A total of 148 instances of civilian protest‐led ousters have been recorded, including the 1989 turnovers in Czechoslovakia and Romania, and the 2011 turnover in Tunisia.^2^

We then transform the upheaval variables across (1) time and (2) space. First, instead of considering an upheaval in a given year, we assign a 1 to country‐years with an upheaval within the past 5 years (and 0 if not). Second, instead of using the three upheaval variables for each country we use a region variable from V‐Dem—dividing the world into 19 distinct regions—to create a new variable capturing all recent upheavals in a given region (which excludes upheavals in the observed country‐year). After transforming the variables, we have far more positive cases: 2631 recent regional revolutions, 5663 recent regional coups, and 1488 recent regional isolated protest‐led ousters.

Our control variables are largely sourced from the V‐Dem dataset. These controls are arranged into four categories. The first group comprises variables related to instability. This encompasses the military dimension index, dummies representing military regimes, along with the number of shocks experienced within the last 5 years as Miller (2021b) underscores the significance of domestic shocks in predicting democratic quality and survival.^3^ These variables were drawn from Geddes et al. (2018) for 1946–2010, and any missing data was supplemented by a recoding of Anckar and Frederiksson (2018) by Miller (2021a, 2021b). Next, we consider economic variables, which might impact democracy; specifically, we include the log of GDP per capita and the rate of economic growth. This data comes from Haber and Menaldo (2011), World Bank (2017), and Gapminder (2018).^4^ The third category involves a demographic variable, the log of the population size as large populations might pose different challenges and dynamics to governance, with data sourced from Gapminder (2018).^5^ Last, we include political factors: the age of the democracy, the count of prior democratic periods, activities indicating corruption, the region's proportion of democracies, and a measure of civil liberties (this refers to the absence of physical violence committed by government agents as well as indicators that reflect government repression that are not directly referring to elections); the age of a democracy and its regional influences can shape its trajectory and robustness (Miller, 2021b). Our controls remain consistent across all models, including model specifications with 5‐year lagged dependent variables (LDVs).

### 4.2 Analytical strategy

Our analytical process aims to better understand the spillover from one country's political instability and to another's democracy and inequality outcomes. The baseline models used across our analyses are identical, each consisting of ordinary least squares (OLS) fixed effects regression with clustered standard errors. In addition to country fixed effects, eliminating biases driven by stable and unobserved heterogeneity between countries (e.g., idiosyncrasies in political systems, fixed cultural differences, or socioeconomic factors), we include year fixed effects to control for time‐varying heterogeneity that is uniform across countries (e.g., global price changes and economic trends or major worldwide events). Including both country and year fixed effects is equivalent to the difference‐in‐difference (DiD) estimator.

There is a debate as to whether researchers should include LDVs (see Achen, 2000; Cook & Webb, 2021; Keele & Kelly, 2006; Plümper et al., 2005; Wilkins, 2018). In time series data, it is often the case that the present value of an observation can be predicted by past values (i.e., autocorrelation). Indeed, this is potentially the case with the equality and democracy data. To control for this, it is sometimes recommended to include lagged values of the dependent variable (Allison, 2009; Beck & Katz, 1995, 1996; De Boef & Keele, 2008; Hicks & Freeman, 2009; Leszczensky & Wolbring, 2019). Others have argued that LDVs ought to be excluded from analyses because they inappropriately suppress the power of other independent variables (Achen, 2000; Plümper et al., 2005), and they have thus been excluded in a good deal of cross‐national comparative research (e.g., Brady et al., 2005; Huber et al., 2008; Jensen & Sørensen, 2012; Huber & Stephens, 2000, 2001, 2012; Huber et al., 2019; Alper et al., 2020; Swank, 2020). Dafoe (2018) presents arguments in favor and against LDVs, stressing that excluding them can create confounding biases and including them can generate collider biases that block the association between a covariate and an outcome. Cook and Webb (2021) argue against the use of LDVs as a general strategy and suggest that they are only appropriate under a restrictive set of assumptions. Because these debates are unresolved, we present our models with and without LDVs, as suggested by Wilkins (2018).

There is also some controversy in the literature around lagged independent variables in cross‐national comparative work, where some researchers favor lagging independent variables and others suggest against the procedure (Bellemare et al., 2017; Brüderl & Ludwig, 2015; Reed, 2015; Vaisey & Miles, 2017). Vaisey and Miles (2017) in particular, point to the difficulties in matching lags in data with lags in underlying causal processes, resulting in potentially highly misleading estimates. Elster (2019) has also registered broader concerns about arbitrary decision‐making when selecting lagged variables.

Miller uses data on social upheavals “in the last 5 years,” and we believe this is a reasonable choice for our analysis too. In our view, a plausible theoretical account proposes that any causal pathway from a revolution in one country to policy change in another will take time, and on these grounds, we use the variables with the original lag structure.

## 5 RESULTS

In Table 1 below we present some basic descriptive statistics on political upheavals and our focal democracy and inequality variables. In each case, we present democracy and inequality measures when there was a recent upheaval in a neighboring country—recent refers to within the past 5 years. We also present those measures for countries at a “baseline,” before there was a recent upheaval. Finally, we look at our measures in the aftermath, 5 years after any recent upheaval.^6^ This allows for a comparison of two before‐after scenarios presented in our table:

(1) the difference between the baseline and a recent upheaval; and
(2) the difference between the baseline and the aftermath

**TABLE 1:** Descriptive statistics on upheavals in the neighborhood.

	Neighbor's recent revolution^a^	Neighbor's recent protest‐led ousters (w/o other shocks)	Neighbor's recent coup
*N*	2631	1488	5663
GDP/capita at baseline (before neighbor's recent upheaval)^b^	$4849	$6757	$4381
GDP/capita today (if neighbor's upheaval is recent)	$5293	$7345	$4714
GDP/capita in aftermath (if neighbor's upheaval is >5 years ago)	$5579	$7278	$5024
% Difference (baseline to today)^c^	9.1%	8.7%	7.6%
% Difference (baseline to well after the upheaval)	15.1%	7.7%	14.7%
GDP growth at baseline (before neighbor's recent upheaval)	1.21%	2.10%	1.56%
GDP growth today (if neighbor's upheaval is recent)	1.24%	1.74%	1.52%
GDP growth in aftermath (if neighbor's upheaval is >5 years ago)	1.54%	1.45%	1.59%
% Difference (baseline to today)	2.8%	−17.4%	−3.0%
% Difference (baseline to well after the upheaval)	27.7%	−31.2%	1.9%
Percent democracy at baseline (before neighbor's recent upheaval)	17.5%	19.5%	19.5%
Percent democracy today (if neighbor's upheaval is recent)	20.1%	22.6%	21.5%
Percent democracy in aftermath (if neighbor's upheaval is >5 years ago)	23.4%	23.0%	21.8%
% Difference (baseline to today)	14.9%	15.9%	10.3%
% Difference (baseline to well after the upheaval)	33.7%	17.9%	11.8%
Electoral democracy index at baseline (before neighbor's recent upheaval)	0.176	0.201	0.190
Electoral democracy index today (if neighbor's upheaval is recent)	0.216	0.249	0.206
Electoral democracy index in aftermath (if neighbor's upheaval is >5 years ago)	0.231	0.249	0.213
% Difference (baseline to today)	22.7%	23.9%	8.4%
% Difference (baseline to well after the upheaval)	31.3%	23.9%	12.1%
Suffrage at baseline (before neighbor's recent upheaval)	0.694	0.715	0.679
Suffrage today (if neighbor's upheaval is recent)	0.719	0.749	0.695
Suffrage in aftermath (if neighbor's upheaval is >5 years ago)	0.733	0.757	0.708
% Difference (baseline to today)	3.6%	4.8%	2.4%
% Difference (baseline to well after the upheaval)	5.6%	5.9%	4.3%
Legal equality at baseline (before neighbor's recent upheaval)	0.554	0.573	0.552
Legal equality today (if neighbor's upheaval is recent)	0.574	0.594	0.564
Legal equality in aftermath (if neighbor's upheaval is >5 years ago)	0.603	0.596	0.570
% Difference (baseline to today)	3.6%	3.7%	2.2%
% Difference (baseline to well after the upheaval)	8.8%	4.0%	3.3%
Egalitarian component index at baseline (before neighbor's recent upheaval)	0.594	0.640	0.578
Egalitarian component index today (if neighbor's upheaval is recent)	0.617	0.657	0.608
Egalitarian component index in aftermath (if neighbor's upheaval is >5 years ago)	0.637	0.659	0.624
% Difference (baseline to today)	3.9%	2.7%	5.2%
% Difference (baseline to well after the upheaval)	7.2%	3.0%	8.0%
Equal distribution at baseline (before neighbor's recent upheaval)	0.510	0.581	0.475
Equal distribution today (if neighbor's upheaval is recent)	0.548	0.600	0.505
Equal distribution in aftermath (if neighbor's upheaval is >5 years ago)	0.570	0.605	0.526
% Difference (baseline to today)	7.5%	3.3%	6.3%
% Difference (baseline to well after the upheaval)	11.7%	4.1%	10.7%

^a^ In this table “recent” refers to a revolution in a neighboring country within the past 5 years.

^b^ GDP/capita is given in real US dollars at the base year 2000. GDP Growth is the 2‐year average change in GDP/capita as a percentage. Data comes from Haber & Menaldo, 2011, World Bank, 2017, and Gapminder, 2018.

^c^ Differences represent the percent change difference.

Both may be worth considering; including the second scenario allows us to capture any delayed responses or lasting changes. For each variable in our table, we present the values at a given time, for example, GDP/capita at the baseline, when an upheaval is in the recent past, and in the aftermath. We then present percent change differences.

We can first describe our democracy variables. For percent democracy, a positive trend was evident across all upheaval scenarios—revolution, isolated protest‐led ousters, and coups in the neighborhood. When we look before and after revolutions transpired in the neighborhood, the countries nearby are more likely to have become democratic. In particular, the growth in percent democracy between the baseline and aftermath was highest when there were nearby revolutions (33.7%), and lowest when there were nearby coups (11.8%).

In terms of the electoral democracy index, all scenarios showed a positive change from baseline to upheaval and baseline to aftermath. Similar to the percent democracy measure, the largest increase in the democracy index was in the case of revolutionary neighbors (31.3%), and again the smallest in the case of coups (12.1%).

With respect to suffrage, there are before‐after differences in all scenarios, and larger ones when we compare the baseline to the aftermath. However, none of the upheaval types show large differences and none stand out from one another. Legal equality increases most in the aftermath of a nearby revolution (8.8%), and least in the aftermath of a nearby coup (3.3%).

The egalitarian component index also saw increases in the context of adjacent transformative mobilizations, with the highest growth observed in aftermath of coups (8.0%) and revolutions (7.2%) and the lowest in the aftermath of isolated protest‐led ousters (3.0%).

Finally, all transformative mobilizations led to an increase in the equal distribution index, with the most significant growth in equality observed when a country had a revolutionary next door (11.7%), and the least with nearby isolated protest‐led ousters (3.3%).

When revolutions and other social upheavals transpire next door, we often see increases in domestic levels of equality and democracy. The suffrage variable is most sensitive to change in the aftermath of isolated protest‐led ousters. The egalitarian component index tends to grow in the aftermath of coups and revolutions. But increases in the remaining variables—percent democracy, electoral democracy, legal equality, and equal distribution—are most likely to be found in the aftermath of a nearby revolution. In general, we observe that when a neighbor has a revolution, we are more likely to see domestic improvements in wellbeing, and these benefits are somewhat less likely in the case of nearby coups.

These descriptive findings are suggestive, but there are reasons to turn to inferential statistics. After all, it could be the case that other changes account for the increase in equality and democracy. Perhaps countries have become more democratic as their income grows. Perhaps democracy and equality grow through autonomous processes over time. The regression analysis that we turn to now includes year‐effects to capture potentially causal time trends and controls for income and a range of other variables to exclude alternative explanations.

**FIGURE 1:**
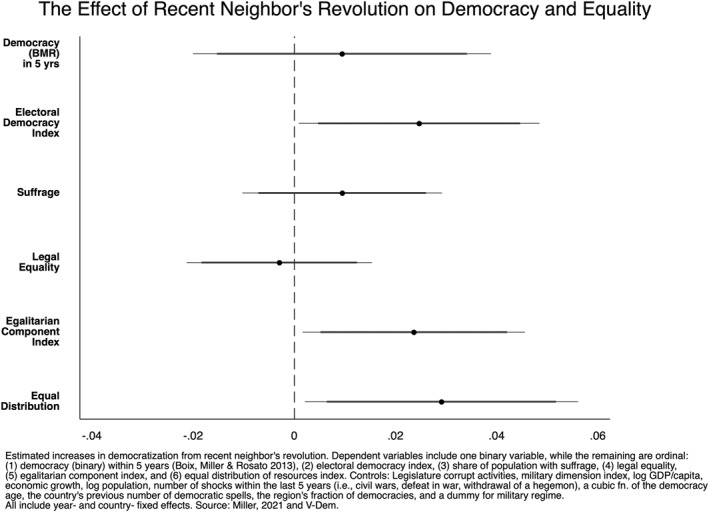
Country‐ and year‐fixed‐effects regressions.

**FIGURE 2:**
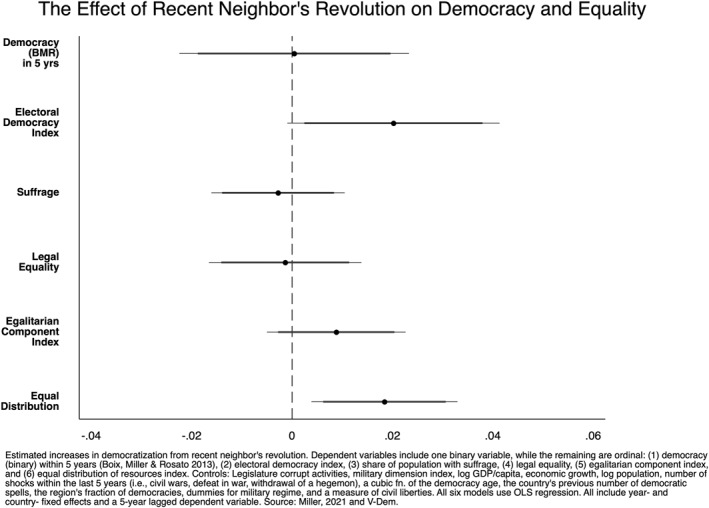
Country‐ and year‐fixed‐effects regressions with 5‐year lagged dependent variable.

**FIGURE 3:**
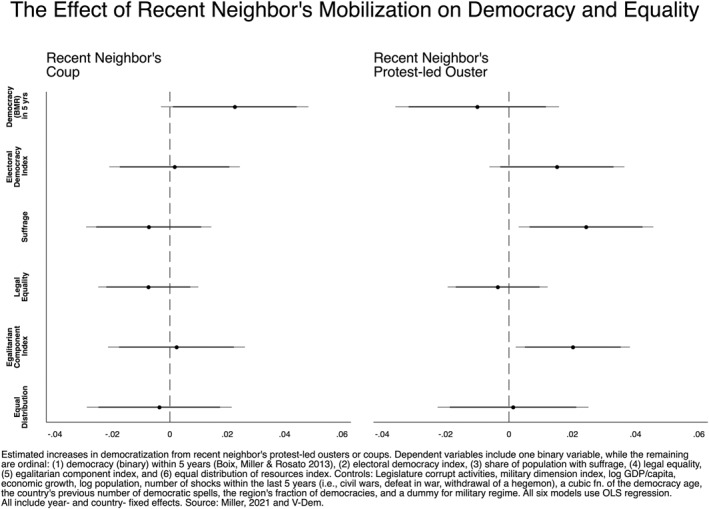
Country‐ and year‐fixed‐effects regressions.

**FIGURE 4:**
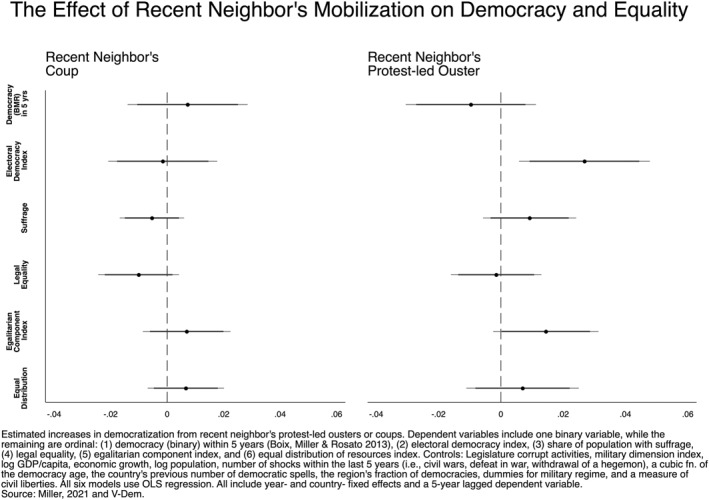
Country‐ and year‐fixed‐effects regressions with 5‐year lagged dependent variable.

We begin our inferential analysis by evaluating the relationship between a recent neighbor's revolution and a range of outcomes. Figures 1, 2, 3, 4 plot the coefficients from our fixed‐effects models to illustrate the effect size of neighboring political upheavals on democracy and inequality (see Appendix for full tables). Figure 1 considers the spillover effects of revolution; Figure 2 replicates the model but includes a 5‐year LDV, as described in our Methods section. Figure 3 considers the spillover effects of coups and isolated protest‐led ousters, and, as above, Figure 4 does the same but includes a 5‐year LDV. The horizontal bars running through each of the six plotted coefficients represent the 90 and 95% confidence intervals (CI). A non‐significant association is reported when a confidence interval crosses the dotted vertical line at 0. The association is positive if the plotted point is to the right of the dotted vertical line and negative when left of the line.

In Figure 1, our model shows statistically significant increases in the electoral democracy index, the egalitarian component index, and the equal distribution index at both 90 and 95% CI, supporting our first hypothesis (H1).^7^ This implies that neighboring countries may experience positive unintended consequences in the form of strengthened democracy and reductions in inequalities following a neighbor's revolution.^8^

However, when we include a 5‐year LDV (5LDV) in Figure 2, the effect of political upheavals diminishes in two of the three significant results. The electoral democracy index remains significant, but only with 90% confidence. The egalitarian component index loses significance. But the equal distribution index remains statistically significant with this more rigorous robustness test.

Turning to the impact of a neighbor's coup, our models in Figure 3 (left side), reveal largely non‐significant results, which aligns with our second hypothesis (H2a). This suggests that coups may not produce any positive effects on foreign democracy and inequality outcomes. The one exception is that our binary measure of democracy shows increases at the 90% confidence level; however, this result does not appear robust to alternative model specification.

When examining the effect of neighbor's recent protest‐led ousters in Figure 3 (right side), the model does show a significant increase in suffrage *and* in the egalitarian component index. The electoral democracy index is non‐significant in this model, although in alternative model specifications it is marginally statistically significant. The remaining measures are non‐significant. This mixed outcome lends partial support to our second sub‐hypothesis (H2b) that these grassroots mobilizations moderately increase democracy and decrease inequality outcomes.

The findings related to the effect of a neighbor's coup remain statistically non‐significant (left side) in the 5LDV model in Figure 4, consistent with the trends in Figure 3. By contrast, the effects of a neighbor's protest‐led ouster (right side) are somewhat less consistent. While the electoral democracy index was non‐significant, it now shows a statistically significant increase. The suffrage measure loses significance. And the egalitarian component index remains significant, but only at a 90% CI. The remainder are consistent with models in Figure 3.

As a whole, our results indicate a dynamic relationship between political upheavals and the ensuing democracy and inequality outcomes in neighboring countries. These findings largely substantiate our hypotheses that revolutions often engender positive changes in democracy and inequality measures, while coups do not. Hypothesis 1 has strong support, as we found statistically significant increases in measures of democracy and inequality following neighboring revolutions. On the other hand, Hypotheses 2a and 2b have varying degrees of support. Hypothesis 2a is supported, as the models reveal no significant impact on democracy or inequality following a neighbor's coup. Hypothesis 2b has partial support, as the results from the protest‐led ousters show a significant, yet mixed, impact on democracy and inequality measures. The effects of isolated protest‐led ousters are only partially consistent, providing some mixed evidence for the idea that such events lead to moderate improvements in democracy and reductions in inequality.

## 6 YOU CAN'T CHOOSE YOUR NEIGHBORS

Are there policy implications that can be drawn out of findings about the activities of your neighbors? From one perspective, it will be hard to learn any lessons at all when the central question concerns politics playing out in a context that is not your own. If we were studying the impact of political upheavals domestically, there would be direct implications for local actors. Domestic actors struggle in their respective countries to generate effective forms of collective action and build internal alliances toward those ends. In other words, we can choose our friends. But we can't choose our neighbors. Strategic conclusions might be elusive when the strategic context is a distant one. We have been studying what are in essence external shocks, and this might limit the quantity and scope of conclusions to glean.

On the other hand, if we are simply trying to understand the world, it is important to recognize external forces that impinge on domestic outcomes. If we learn that child development has a stronger than expected genetic component, it informs parents about the scope of their control. Some outcomes are responsive to policy, others fall outside its purview, and effective social policymaking requires the wisdom to know the difference. Social change will often operate behind the backs of actors, and understanding it entails understanding intended as well as unintended forces.

Nonetheless, there are lessons to be drawn, but since there are different interests in play, any lessons will depend on the actor in question. For example, there are different implications for political elites and ordinary citizens. Elites can gain insight into the types of foreign events that should warrant their caution, potentially even adopting pre‐emptive measures to mitigate the risk of such events. From the perspective of elites hoping to limit redistribution or reduce any loss of political control, nearby revolutions are far more concerning than coups and perhaps also isolated protest‐led ousters. Nearby coups might be more ignorable than revolutionary moments. By contrast, devoting resources to neighboring countries' efforts in undermining revolutionary threats could be worthwhile from the perspective of domestic elites trying to retain power and resources. From their perspective, a neighborhood watch association might be valuable.

It is worth speculating on why protest‐led ousters may not translate as strongly into regional elite fears when we compare with the impact of revolution. It could be that the level of organization and military capacity of rebels simply poses a greater threat. It may also be the case that the downstream consequences of a revolution are worse for elites than those of a protest‐led ouster. Ousted elites may be more likely to survive in the latter context.^9^

Consider now, ordinary citizens and domestic social reformers, who might take a greater interest in revolutionary moments in bordering nations. There is, of course, a standard collective action problem; after all, for a given external group, any benefits can be garnered without contributing to the revolution next door (Hardin, 1982; Offe & Wiesenthal, 1980; Olsen, 1965). Nonetheless, domestic social movements might find that revolutions at a distance are preferable to those at home, and at minimum, if our findings are correct, revolutionary neighbors could merit support.

Becoming more international in scope might be a strategy social movements take more seriously, at least those in contexts where regional revolutionary moments are still on the table. This conclusion would align social movements with insights from traditions that have been far more international in orientation going back more than a century (Sassoon, 1998). Actors advocating for democracy and equality as well as those working to impede its expansion should therefore evaluate the pros and cons of fostering or frustrating these cross‐border relationships.

There is a related question: do the effects we find in the paper operate in non‐democratic contexts only? One might speculate that democracies already have institutions that can channel popular demands, which is one of the variables that takes revolution off the table and might lead domestic socialists to parliamentary strategies at home (Calnitsky, 2022). We rerun the analysis after excluding democracies and find that one of our equality measures remains strongly significant and the other becomes marginally non‐significant. The electoral democracy measure, however, loses significance. Some of this effect may be due to a lower observation count, given the broad definition of democracy from Boix et al. (2013). This also suggests that nominal democracies are themselves at least sometimes influenced by neighboring revolutions. The effects we observe may sometimes be in play in nominally democratic settings, given the broad definition, as well as autocratic ones. Relatedly, a measure of government quality or effectiveness may play a role because well‐governed regimes may be more secure. We use a variable for “regime corruption” (V‐Dem) and rerun the analysis after excluding high corruption (i.e., above the median) cases, and we find that less corrupt regimes may be insulated from the effects we observe; our models with electoral democracy and our two equality measures lose significance. Local activists may therefore be less sanguine about domestic spillovers in these cases. Finally, “coercive capacity” may be a way to insulate from the effects too, and high GDP might proxy for an ability to fund repressive responses to threats. Again, when we exclude low GDP cases—keeping only those above the median—our variables lose statistical significance. Of course, this may capture other mechanisms—resources facilitate both coercion and consent—but, speculatively, it is plausible that the relationship we observe plays out largely in contexts that are poorer and have lower capacities for coercion. In contexts with more resources and more expansive democracies, reformers and socialists abandoned the revolution and shifted their struggles to parliament (Calnitsky & Wind, 2024); they might also be at least somewhat less hopeful that outside revolution redound to their benefit at home.

The debate among activists about internationalism and cross‐border influences, as noted above, is an old one. Perhaps the most acrimonious debate in the history of the left was essentially about spillover effects. Again, Stalin argued for their irrelevance, while Trotsky waged all his political credibility on it (Kolakowski, 1978; Van Ree, 1998). The major policy question was about the relevance of revolutionary spillover effects for the stability of the country where the revolution originally took place. The core idea was that for a revolution to survive, there had to be spillovers.^10^ The debate itself was politically charged and intense enough to cause bloodshed, but underlying that debate was an empirical claim about the world: is there a causal mechanism linking revolution in one country to revolution elsewhere? The stakes were high because of the implications for the success or failure of revolution. This paper has explored a related question, asking if revolution in one country can spark reform in another. The stakes are similarly high, if revolutionary threats are indeed central to the success of egalitarian and democratic reforms.

When we consider the road from revolution at home to reform abroad, the policy conclusions might in fact run *opposite* to those hypothesized in the debate above. If a revolution in one country foments reform in another, the core Troskyist hypothesis is weakened. What is happening is that revolution in one country—by increasing the odds of reform in another—is actually undermining the social bases of revolution and therefore the likelihood that it spreads around the world. In other words, there very well may be some mechanisms whereby revolution is self‐defeating. If revolution sparks revolution this is not the case, but if it sparks reform, it is.

For revolutionaries, the conclusion that revolution fosters reform might not be a welcome one. But the conclusions could be uncomfortable for reformists too. Peaceful reformers may, at certain times and places, benefit from alliances with revolutionaries. The underlying mechanism might suggest, for example, that Martin Luther King can benefit from the very existence of Malcolm X. The former might be more effective when the threat of the latter is present. The effectiveness of Gandhi's peaceful forms of collective action might have benefited from the fact that they emerged in the context of violent riots, and he was seen as the more reasonable alternative. Wasow (2020) analyzed the relative efficacy of violent and non‐violent mobilizations, but not the relationship between them. The juxtaposition of violent and peaceful forms of change might encourage the wider acceptance of more peaceful methods, viewed as the lesser of two evils.

Yet, the presence of revolutionary neighbors can also have adverse effects. The spread of revolutionary sentiments may create social instability, contribute to political fragmentation, and cause humanitarian crises. Based on Trotsky's criticism of Stalin's “socialism in one country”, the adverse impacts could be exacerbated if a revolution in one country results in a parasitic bureaucracy in another (Kolakowski, 1978). We have, moreover, not tested whether revolution in one country generates the same in another, and even if revolution is far less common on average than reform, the potential for a direct copycat effect might be part of the calculation too.

So, should movements for social change endorse or renounce their revolutionary neighbors? A revolution next door may have significant advantages for a transformative agenda. It could ignite the spark of collective action domestically, invigorate the local working classes, and foster solidarity among social movements across borders. It is worth noting that we have presented an empirical relationship but have not presented any evidence in favor of one mechanism or another. If revolutionary neighbors foster equality, it could be because they galvanize workers or ethnic movements. But they might not. Violent upheavals might just instill fear in elites. Or, they might even instill foresight in elites. Nonetheless, it seems reasonable to expect that revolutionary neighbors could serve as a beacon of hope for social movements in countries where the possibility of a revolution seems remote or unlikely.

On the other hand, the prospect of revolutionary neighbors may present considerable challenges to movements for social change. The spread of revolutionary sentiment might lead to internal divisions, cause violent conflicts, and even provoke foreign interventions. And even if positive equality effects hold on average, the possibility of repression could be less likely but more severe when it does emerge. The same could be true for any democracy effects; an authoritarian response might be rare, but when it transpires, it could be extreme.

Our study describes a complex relationship between regional and local events. Most cross‐national time series analyses describe different countries as entirely separate units to be pooled and aggregated together. The methodological assumptions of regression analysis typically require that observations are independent of one another. As such, analyses of various countries over time treat all relationships between variables as *within* country relationships. But we know that a variable in one country can impact a different one in another country. While cross‐national time series analysis often ignores this reality, the field of International Relations takes it as a methodological premise. And World Systems Theory takes the premise to an extreme conclusion, arguing that all cross‐national analysis ought to collapse into a single unit, the world (Wallerstein, 1979). This is almost certainly wrong: while cross border effects exist, causal social mechanisms are also pervasive at the national level. And while cross‐national influences might violate some of the strict assumptions of regression analysis, it ought not lead researchers to abandon all quantitative cross‐national time series analysis. It instead ought to serve as a reminder that cross‐national data can draw out relationships between, as well as within, countries. It can help us explain the mainsprings of transformative processes at the national level.

Our understanding of social change is significantly deepened by scrutinizing the dynamic relationship between regional and local phenomena. Analyzing regional events and domestic transformations allows us to better appreciate the influences that revolutionary neighbors can exert. The bloody debate over “socialism in one country” captures the stakes inherent in these interactions. When domestic events are massive there is no reason to assume they will be contained inside borders. But the moral implications are far from straightforward. On normative grounds, we believe that the insights presented in this paper are double‐edged: they have the potential to serve those who aspire to change the world, but they could just as easily be leveraged by actors invested in obstructing such changes.

## CONFLICT OF INTEREST STATEMENT

There is no conflict of interest.

## Supporting information

Supporting Information S1

## Data Availability

The data supporting the findings of this paper are openly available at the Varieties of Democracy database [https://v‐dem.net/] at doi.org/10.23696/vdemds23 and *Shock to the System* data [https://sites.google.com/site/mkmtwo/data].

## ENDNOTES

^1^
Depending on the analysis, data either goes back to 1800 or 1900.

^2^
Following Miller, data for protest‐led ousters exclude those cases where there are other major “shocks” occurring at the same time.

^3^
The military dimension index asks about the extent to which the power base of the chief executive is determined by the military. The index is based on whether the “chief executive” was (a) appointed through a coup, rebellion or by the military, and (b) can be dismissed by the military. Both condition (a) and (b) are coded as present (1) or not (0); and the variable then averages across the two. The “shocks” variable limits the definition to five of the most significant violent events, divided into domestic elite conflicts (coups, civil wars, and assassinations) and foreign shocks (defeat in war and withdrawal of an autocratic hegemon).

^4^
It may also be worth saying something about our two economic control variables and their association with political upheavals. Starting with GDP per capita, we see increases across all upheaval scenarios. The largest rise is observed in countries that experienced a recent revolution (15.1 percent), while the aftermath of isolated protest‐led ousters in neighboring countries without other shocks resulted in a modest increase of 7.7 percent. In contrast, GDP growth patterns are more mixed. The aftermath of a neighbor's recent revolution leads to 27.7 percent growth, meanwhile coups in neighbors only produce a marginal 1.9 percent uptick. Interestingly, GDP growth in countries with isolated protest‐led ousters decreases by 31.2 percent. While all neighboring political upheaval scenarios elevate GDP per capita, GDP growth effects vary, with revolutions in neighboring countries typically providing the most substantial economic boosts.

^5^
Missing values for this group were substituted by data from CNTS for the years 1815–1939, and Heston et al. (2011) for subsequent years.

^6^
To clarify, our independent variables refer to regional revolutions, coups, or protest‐led ousters in the last 5 years. The measures “at the baseline” considers data 5 years prior, that is, we lag the variable by 5 years, to capture outcomes before any upheaval took place. And the “aftermath” measures refer to a 5‐year lead on the variables. Miller (2021b) underscores the significant influence of shocks, such as coups and hegemonic withdrawals, on democratization trajectories. These shocks intensify regime vulnerability and escalate violent challenges like coups and civil conflicts. To accurately gauge the effects of protest‐led ousters, it is essential to distinguish them from these overarching shocks. Including them together could conflate their effects, compromising the clarity of the regression analysis. Miller emphasizes that without recent shocks, protest‐led ousters do not necessarily herald democratization. Thus, to maintain analytical precision, our focus remains only on isolated protest‐led ousters, excluding concurrent major shocks.

^7^
It is worth mentioning here the literature on two‐way fixed effects and the scenarios of “forbidden comparisons” and negative weights (De Chaisemartin & d’Haultfoeuille, 2020; Roth et al., 2023). One of the main worries is the possibility of results with the opposite sign from what it ought to be. As a simple solution, we have rerun the analysis first using only year fixed effects, then only country fixed effects, and then with neither. Our signs do not change. But we have also run some basic diagnostics suggested in the literature (Roth et al., 2023), in particular we check for the presence of negative weights in the case of our main regressions using the “twowayfeweights” command in Stata; the results give us a picture of the overall weight distribution. In nearly all cases, weights are positive, and the total sum of negative weights is effectively zero. While the presence of any negative weights can be a concern, the impact here is minimal, suggesting these few cases do not significantly distort the overall estimation.

^8^
We have also tested the robustness of the analysis with a range of other permutations of controls—always, however, keeping our economic and demographic variables in place—and our results are consistent. In all cases “equal distribution” and “egalitarian component” remain significant, but in a minority of cases the “electoral democracy” measure loses significance. Following Miller we include additional tests with a variable capturing “personalist regime” (Miller extends this variable created by Geddes et al., 2018) and one capturing “gender power equity” from the Varieties of Democracy database; results are virtually identical to those presented in the figures (results available on request).

^9^
Consider a few examples. In the case of the 2011 Libyan revolution, the level of organization and military capacity of the rebels played a crucial role; they turned the upheaval into an armed conflict against Gaddafi's regime, which led to his killing. This revolution not only prompted the overthrow of the regime but also resulted in significant regional repercussions, including heightened concerns among neighboring countries' elites about similar uprisings. A contrasting case is the 1986 People Power Revolution in the Philippines, where ousted elites managed to survive and maintain some influence. Unlike the violent aftermath of many revolutions, this largely peaceful protest‐led ouster resulted in the exile of Marcos, but did not completely dismantle the existing elite structure; Marcos and his allies retained some level of influence and resources, which contrasts with the more definitive elite displacement often seen in full‐scale revolutions. Similarly, where the Iranian Revolution in 1979 serves as an example of severe downstream consequences for elites, the ousted elites of the Color Revolutions in Eastern Europe often remained alive, sometimes even retaining political influence or making comebacks in later years.

^10^
It is of course true that neither party in the debate rejected the idea of revolution, and it is also true that the question of exporting the revolution was downstream from the bigger question of the development of socialism in a poor country. Nonetheless, the analogy to the question in this paper remains, and it is notable that a whole generation of activists on the left felt the need to take strong positions on a question that comes down to spillover effects.
