# Supplementary material for: The revolution next door
Source: Br J Sociol. 2024 Jul 8;75(5):769–90. doi: 10.1111/1468-4446.13131 (PMC11617795; doi:10.1111/1468-4446.13131)
Supplement: Supplementary file 1 — Supporting Information S1 [file BJOS-75-769-s001.docx]

**Appendix. Country- and year-fixed-effects model tables**

**Table A1. Country- and year-fixed-effects models democracy and inequality on revolution**

|  | (1) | (2) | (3) | (4) | (5) | (6) |
| --- | --- | --- | --- | --- | --- | --- |
|  | Democracy in 5 yrs. | Electoral Democracy | Suffrage | Legal Equality | Egalitarian Component | Equal Distribution |
|  |  | Index |  |  | Index |  |
|  |  |  |  |  |  |  |
| Recent Neighbor’s Revolution | 0.00941 | 0.0246** | 0.00945 | -0.00298 | 0.0236** | 0.0291** |
|  | (0.0150) | (0.0121) | (0.0101) | (0.00935) | (0.0112) | (0.0138) |
| Legislature Corruption | 0.0374*** | 0.0497*** | 0.0230* | -0.0178* | 0.0483*** | 0.0367** |
|  | (0.0129) | (0.0120) | (0.0121) | (0.0101) | (0.0151) | (0.0164) |
| Military Dimension Index | -0.0809* | -0.118** | 0.0359 | 0.0949** | -0.0287 | -0.127 |
|  | (0.0418) | (0.0456) | (0.0468) | (0.0432) | (0.0697) | (0.0854) |
| GDP/Capita | 0.00797 | 0.0258 | -0.0482* | -0.0387** | -0.0555* | 0.0136 |
|  | (0.0241) | (0.0222) | (0.0253) | (0.0184) | (0.0317) | (0.0334) |
| Economic Growth | -0.000931 | -0.000348 | 0.000331 | 0.00127** | 0.000641 | 0.000234 |
|  | (0.000699) | (0.000626) | (0.000590) | (0.000498) | (0.000693) | (0.000855) |
| Population | 0.0286 | 0.0121 | 0.0324 | 0.105*** | 0.147*** | 0.0787 |
|  | (0.0357) | (0.0306) | (0.0294) | (0.0276) | (0.0387) | (0.0537) |
| Number of Shocks (Past 5 yrs.) | 0.0158 | -0.000458 | 0.00653 | -0.0117 | 0.00363 | -0.00438 |
|  | (0.0113) | (0.00677) | (0.00952) | (0.00761) | (0.0116) | (0.0121) |
| Regional Democracy | -0.0856 | 0.0761 | 0.0246 | 0.0373 | -0.0668 | -0.118 |
|  | (0.0601) | (0.0606) | (0.0693) | (0.0491) | (0.0834) | (0.0932) |
| Past Democratic Spells | 0.0623** | 0.0617** | 0.0272 | -0.0306 | 0.0213 | 0.0410 |
|  | (0.0284) | (0.0275) | (0.0248) | (0.0236) | (0.0282) | (0.0406) |
| Democracy Age | -0.00627*** | -0.000915 | -0.000327 | -0.00175* | -0.000142 | 0.00198 |
|  | (0.00121) | (0.00106) | (0.00146) | (0.00105) | (0.00144) | (0.00151) |
| Democracy Age Squared | 3.54e-05** | -6.07e-06 | -2.46e-05 | 1.18e-05 | -2.05e-05 | -4.51e-05** |
|  | (1.41e-05) | (1.39e-05) | (1.94e-05) | (1.47e-05) | (2.00e-05) | (2.00e-05) |
| Democracy Age Cubic | -1.14e-07** | 3.26e-08 | 1.32e-07* | -2.68e-08 | 9.86e-08 | 1.79e-07** |
|  | (5.05e-08) | (5.02e-08) | (7.27e-08) | (5.81e-08) | (7.97e-08) | (7.43e-08) |
| Military Regime | 0.0129 | -0.0170 | 0.0317 | -0.0209 | -0.0264 | -0.0270 |
|  | (0.0274) | (0.0213) | (0.0312) | (0.0230) | (0.0345) | (0.0353) |
| Civil Liberties | 0.758*** | 0.859*** | -0.0302 | 1.193*** | 0.228*** | 0.0671 |
|  | (0.0788) | (0.0547) | (0.0598) | (0.0513) | (0.0728) | (0.0832) |
|  |  |  |  |  |  |  |
| Observations | 12,078 | 11,977 | 12,079 | 12,079 | 10,070 | 10,070 |
| R-squared | 0.620 | 0.665 | 0.680 | 0.632 | 0.466 | 0.378 |
| Number of Countries | 171 | 171 | 171 | 171 | 171 | 171 |
| Country FE? | YES | YES | YES | YES | YES | YES |
| Year FE? | YES | YES | YES | YES | YES | YES |

Robust standard errors in parentheses

*** p<0.01, ** p<0.05, * p<0.1

**Table A2. Country- and year-fixed-effects models democracy and inequality on revolution (5-year LDV)**

|  | (1) | (2) | (3) | (4) | (5) | (6) |
| --- | --- | --- | --- | --- | --- | --- |
|  | Democracy in 5 yrs. | Electoral Democracy | Suffrage | Legal Equality | Egalitarian Component | Equal Distribution |
|  |  | Index |  |  | Index |  |
|  |  |  |  |  |  |  |
| Recent Neighbor’s Revolution | 0.00627 | 0.00842 | 0.00133 | -0.00929 | 0.0245** | 0.0264* |
|  | (0.0176) | (0.0138) | (0.0101) | (0.0116) | (0.0113) | (0.0137) |
| Legislature Corruption | 0.0330** | 0.0362*** | 0.0226* | -0.0202* | 0.0386*** | 0.0347** |
|  | (0.0151) | (0.0120) | (0.0117) | (0.0109) | (0.0141) | (0.0158) |
| Military Dimension Index | -0.0559 | -0.136*** | 0.0291 | 0.0749* | -0.0190 | -0.0912 |
|  | (0.0494) | (0.0462) | (0.0446) | (0.0438) | (0.0582) | (0.0772) |
| GDP/Capita | 0.00573 | 0.0425* | -0.0473* | -0.0506** | -0.0462 | 0.00776 |
|  | (0.0283) | (0.0236) | (0.0273) | (0.0210) | (0.0348) | (0.0344) |
| Economic Growth | 0.000403 | -0.000401 | 0.000375 | 0.00133* | 0.000323 | -0.000198 |
|  | (0.000649) | (0.000653) | (0.000624) | (0.000677) | (0.000568) | (0.000651) |
| Population | 0.0424 | 0.0223 | 0.0264 | 0.105*** | 0.153*** | 0.0726 |
|  | (0.0380) | (0.0316) | (0.0298) | (0.0300) | (0.0400) | (0.0549) |
| Number of Shocks (Past 5 yrs.) | 0.00681 | 0.00721 | 0.00834 | -0.00209 | 0.00131 | -0.00662 |
|  | (0.0127) | (0.00766) | (0.00917) | (0.00870) | (0.0111) | (0.0109) |
| Regional Democracy | -0.0372 | 0.0768 | 0.0215 | 0.0510 | -0.0595 | -0.112 |
|  | (0.0710) | (0.0637) | (0.0700) | (0.0516) | (0.0782) | (0.0912) |
| Past Democratic Spells | -0.00160 | 0.0344 | 0.0206 | -0.0408 | 0.0292 | 0.0512 |
|  | (0.0291) | (0.0247) | (0.0228) | (0.0282) | (0.0285) | (0.0427) |
| Democracy Age | -0.00693*** | -0.00177 | -0.000905 | -0.00285** | -0.000238 | 0.00185 |
|  | (0.00149) | (0.00129) | (0.00151) | (0.00120) | (0.00148) | (0.00149) |
| Democracy Age Squared | 4.57e-05** | 3.32e-06 | -1.67e-05 | 2.26e-05 | -1.68e-05 | -4.23e-05** |
|  | (1.87e-05) | (1.64e-05) | (2.04e-05) | (1.66e-05) | (2.04e-05) | (2.01e-05) |
| Democracy Age Cubic | -1.57e-07** | -5.61e-09 | 1.07e-07 | -5.89e-08 | 8.68e-08 | 1.73e-07** |
|  | (6.98e-08) | (5.92e-08) | (7.74e-08) | (6.54e-08) | (8.00e-08) | (7.53e-08) |
| Military Regime | 0.0155 | 0.0297 | 0.0298 | -0.0121 | -0.0391 | -0.0373 |
|  | (0.0295) | (0.0213) | (0.0320) | (0.0243) | (0.0310) | (0.0293) |
| Civil Liberties | 0.586*** | 0.688*** | -0.0407 | 0.917*** | 0.162** | 0.0441 |
|  | (0.0813) | (0.0577) | (0.0577) | (0.0529) | (0.0685) | (0.0805) |
|  |  |  |  |  |  |  |
| Observations | 11,444 | 11,360 | 11,445 | 11,445 | 9,592 | 9,592 |
| R-squared | 0.531 | 0.588 | 0.687 | 0.471 | 0.447 | 0.367 |
| Number of Countries | 170 | 170 | 170 | 170 | 170 | 170 |
| Country FE? | YES | YES | YES | YES | YES | YES |
| Year FE? | YES | YES | YES | YES | YES | YES |

Robust standard errors in parentheses

*** p<0.01, ** p<0.05, * p<0.1

**Table A3. Country- and year-fixed-effects models democracy and inequality on protest-led ousters**

|  | (1) | (2) | (3) | (4) | (5) | (6) |
| --- | --- | --- | --- | --- | --- | --- |
|  | Democracy in 5 yrs. | Electoral Democracy | Suffrage | Legal Equality | Egalitarian Component | Equal Distribution |
|  |  | Index |  |  | Index |  |
|  |  |  |  |  |  |  |
| Recent Neighbor’s Ouster | -0.0100 | 0.0151 | 0.0243** | -0.00357 | 0.0202** | 0.00128 |
|  | (0.0132) | (0.0109) | (0.0108) | (0.00806) | (0.00920) | (0.0122) |
| Legislature Corruption | 0.0369*** | 0.0487*** | 0.0227* | -0.0177* | 0.0472*** | 0.0353** |
|  | (0.0128) | (0.0121) | (0.0120) | (0.0101) | (0.0150) | (0.0164) |
| Military Dimension Index | -0.0811* | -0.119** | 0.0358 | 0.0949** | -0.0291 | -0.127 |
|  | (0.0418) | (0.0457) | (0.0468) | (0.0432) | (0.0695) | (0.0855) |
| GDP/Capita | 0.00797 | 0.0251 | -0.0487* | -0.0386** | -0.0565* | 0.0132 |
|  | (0.0241) | (0.0221) | (0.0252) | (0.0184) | (0.0318) | (0.0336) |
| Economic Growth | -0.000960 | -0.000377 | 0.000338 | 0.00128** | 0.000621 | 0.000179 |
|  | (0.000694) | (0.000632) | (0.000593) | (0.000498) | (0.000689) | (0.000854) |
| Population | 0.0283 | 0.0109 | 0.0317 | 0.105*** | 0.145*** | 0.0779 |
|  | (0.0357) | (0.0306) | (0.0294) | (0.0276) | (0.0388) | (0.0536) |
| Number of Shocks (Past 5 yrs.) | 0.0159 | -8.81e-05 | 0.00668 | -0.0118 | 0.00421 | -0.00380 |
|  | (0.0112) | (0.00676) | (0.00953) | (0.00761) | (0.0117) | (0.0120) |
| Regional Democracy | -0.0856 | 0.0742 | 0.0231 | 0.0376 | -0.0707 | -0.121 |
|  | (0.0599) | (0.0607) | (0.0689) | (0.0492) | (0.0836) | (0.0937) |
| Past Democratic Spells | 0.0622** | 0.0611** | 0.0268 | -0.0305 | 0.0208 | 0.0406 |
|  | (0.0284) | (0.0277) | (0.0249) | (0.0236) | (0.0284) | (0.0408) |
| Democracy Age | -0.00623*** | -0.000918 | -0.000375 | -0.00174* | -0.000153 | 0.00201 |
|  | (0.00121) | (0.00106) | (0.00146) | (0.00104) | (0.00145) | (0.00151) |
| Democracy Age Squared | 3.49e-05** | -5.92e-06 | -2.39e-05 | 1.17e-05 | -2.03e-05 | -4.53e-05** |
|  | (1.41e-05) | (1.38e-05) | (1.94e-05) | (1.47e-05) | (2.00e-05) | (2.00e-05) |
| Democracy Age Cubic | -1.13e-07** | 3.18e-08 | 1.29e-07* | -2.65e-08 | 9.72e-08 | 1.79e-07** |
|  | (5.04e-08) | (4.99e-08) | (7.26e-08) | (5.80e-08) | (7.96e-08) | (7.44e-08) |
| Military Regime | 0.0123 | -0.0172 | 0.0321 | -0.0209 | -0.0262 | -0.0279 |
|  | (0.0274) | (0.0214) | (0.0312) | (0.0231) | (0.0343) | (0.0349) |
| Civil Liberties | 0.757*** | 0.857*** | -0.0300 | 1.194*** | 0.227*** | 0.0648 |
|  | (0.0786) | (0.0550) | (0.0601) | (0.0513) | (0.0734) | (0.0838) |
|  |  |  |  |  |  |  |
| Observations | 12,078 | 11,977 | 12,079 | 12,079 | 10,070 | 10,070 |
| R-squared | 0.620 | 0.665 | 0.680 | 0.632 | 0.465 | 0.376 |
| Number of Countries | 171 | 171 | 171 | 171 | 171 | 171 |
| Country FE? | YES | YES | YES | YES | YES | YES |
| Year FE? | YES | YES | YES | YES | YES | YES |

Robust standard errors in parentheses

*** p<0.01, ** p<0.05, * p<0.1

**Table A4. Country- and year-fixed-effects models democracy and inequality on protest-led ousters (5-year LDV)**

|  | (1) | (2) | (3) | (4) | (5) | (6) |
| --- | --- | --- | --- | --- | --- | --- |
|  | Democracy in 5 yrs. | Electoral Democracy | Suffrage | Legal Equality | Egalitarian Component | Equal Distribution |
|  |  | Index |  |  | Index |  |
|  |  |  |  |  |  |  |
| Recent Neighbor’s Ouster | -0.0106 | 0.0181 | 0.0227* | -0.00517 | 0.0232** | -0.00453 |
|  | (0.0178) | (0.0143) | (0.0117) | (0.0115) | (0.0117) | (0.0137) |
| Legislature Corruption | 0.0328** | 0.0358*** | 0.0225* | -0.0198* | 0.0374*** | 0.0336** |
|  | (0.0151) | (0.0120) | (0.0117) | (0.0109) | (0.0141) | (0.0158) |
| Military Dimension Index | -0.0559 | -0.135*** | 0.0293 | 0.0748* | -0.0183 | -0.0917 |
|  | (0.0494) | (0.0462) | (0.0444) | (0.0438) | (0.0581) | (0.0774) |
| GDP/Capita | 0.00618 | 0.0421* | -0.0480* | -0.0506** | -0.0466 | 0.00884 |
|  | (0.0283) | (0.0235) | (0.0272) | (0.0211) | (0.0348) | (0.0347) |
| Economic Growth | 0.000396 | -0.000417 | 0.000368 | 0.00134** | 0.000287 | -0.000235 |
|  | (0.000651) | (0.000655) | (0.000625) | (0.000676) | (0.000568) | (0.000654) |
| Population | 0.0421 | 0.0217 | 0.0262 | 0.106*** | 0.151*** | 0.0715 |
|  | (0.0381) | (0.0316) | (0.0299) | (0.0300) | (0.0400) | (0.0546) |
| Number of Shocks (Past 5 yrs.) | 0.00690 | 0.00715 | 0.00825 | -0.00213 | 0.00146 | -0.00639 |
|  | (0.0127) | (0.00766) | (0.00916) | (0.00872) | (0.0111) | (0.0109) |
| Regional Democracy | -0.0370 | 0.0775 | 0.0218 | 0.0505 | -0.0579 | -0.111 |
|  | (0.0709) | (0.0635) | (0.0700) | (0.0515) | (0.0785) | (0.0918) |
| Past Democratic Spells | -0.00191 | 0.0342 | 0.0207 | -0.0405 | 0.0284 | 0.0501 |
|  | (0.0292) | (0.0248) | (0.0229) | (0.0282) | (0.0289) | (0.0428) |
| Democracy Age | -0.00690*** | -0.00179 | -0.000945 | -0.00286** | -0.000236 | 0.00187 |
|  | (0.00148) | (0.00128) | (0.00151) | (0.00120) | (0.00148) | (0.00149) |
| Democracy Age Squared | 4.52e-05** | 3.76e-06 | -1.60e-05 | 2.27e-05 | -1.66e-05 | -4.27e-05** |
|  | (1.87e-05) | (1.64e-05) | (2.04e-05) | (1.66e-05) | (2.05e-05) | (2.01e-05) |
| Democracy Age Cubic | -1.55e-07** | -7.51e-09 | 1.04e-07 | -5.88e-08 | 8.56e-08 | 1.75e-07** |
|  | (6.96e-08) | (5.91e-08) | (7.74e-08) | (6.54e-08) | (8.02e-08) | (7.55e-08) |
| Military Regime | 0.0150 | 0.0296 | 0.0301 | -0.0117 | -0.0400 | -0.0391 |
|  | (0.0296) | (0.0212) | (0.0318) | (0.0245) | (0.0309) | (0.0291) |
| Civil Liberties | 0.584*** | 0.689*** | -0.0383 | 0.918*** | 0.162** | 0.0402 |
|  | (0.0809) | (0.0575) | (0.0579) | (0.0531) | (0.0689) | (0.0811) |
|  |  |  |  |  |  |  |
| Observations | 11,444 | 11,360 | 11,445 | 11,445 | 9,592 | 9,592 |
| R-squared | 0.531 | 0.588 | 0.687 | 0.471 | 0.446 | 0.366 |
| Number of Countries | 170 | 170 | 170 | 170 | 170 | 170 |
| Country FE? | YES | YES | YES | YES | YES | YES |
| Year FE? | YES | YES | YES | YES | YES | YES |

Robust standard errors in parentheses

*** p<0.01, ** p<0.05, * p<0.1

**Table A5. Country- and year-fixed-effects models democracy and inequality on coups**

|  | (1) | (2) | (3) | (4) | (5) | (6) |
| --- | --- | --- | --- | --- | --- | --- |
|  | Democracy in 5 yrs. | Electoral Democracy | Suffrage | Legal Equality | Egalitarian Component | Equal Distribution |
|  |  | Index |  |  | Index |  |
|  |  |  |  |  |  |  |
| Recent Neighbor’s Coup | 0.0226* | 0.00163 | -0.00737 | -0.00752 | 0.00230 | -0.00370 |
|  | (0.0131) | (0.0116) | (0.0111) | (0.00890) | (0.0122) | (0.0129) |
| Legislature Corruption | 0.0382*** | 0.0487*** | 0.0222* | -0.0181* | 0.0474*** | 0.0350** |
|  | (0.0128) | (0.0120) | (0.0121) | (0.0100) | (0.0151) | (0.0163) |
| Military Dimension Index | -0.0801* | -0.119** | 0.0354 | 0.0946** | -0.0295 | -0.127 |
|  | (0.0414) | (0.0458) | (0.0469) | (0.0431) | (0.0697) | (0.0855) |
| GDP/Capita | 0.00752 | 0.0254 | -0.0483* | -0.0386** | -0.0559* | 0.0134 |
|  | (0.0239) | (0.0222) | (0.0254) | (0.0183) | (0.0320) | (0.0336) |
| Economic Growth | -0.000916 | -0.000389 | 0.000302 | 0.00127** | 0.000598 | 0.000173 |
|  | (0.000699) | (0.000631) | (0.000591) | (0.000496) | (0.000694) | (0.000852) |
| Population | 0.0270 | 0.0110 | 0.0323 | 0.106*** | 0.146*** | 0.0785 |
|  | (0.0356) | (0.0305) | (0.0295) | (0.0276) | (0.0388) | (0.0534) |
| Number of Shocks (Past 5 yrs.) | 0.0165 | -8.74e-05 | 0.00649 | -0.0120 | 0.00413 | -0.00385 |
|  | (0.0113) | (0.00677) | (0.00953) | (0.00756) | (0.0117) | (0.0120) |
| Regional Democracy | -0.0848 | 0.0749 | 0.0237 | 0.0371 | -0.0689 | -0.122 |
|  | (0.0602) | (0.0606) | (0.0691) | (0.0493) | (0.0838) | (0.0941) |
| Past Democratic Spells | 0.0629** | 0.0614** | 0.0268 | -0.0308 | 0.0211 | 0.0404 |
|  | (0.0281) | (0.0277) | (0.0249) | (0.0235) | (0.0283) | (0.0408) |
| Democracy Age | -0.00614*** | -0.000872 | -0.000355 | -0.00179* | -0.000108 | 0.00199 |
|  | (0.00122) | (0.00106) | (0.00144) | (0.00105) | (0.00145) | (0.00152) |
| Democracy Age Squared | 3.39e-05** | -6.52e-06 | -2.43e-05 | 1.23e-05 | -2.09e-05 | -4.51e-05** |
|  | (1.43e-05) | (1.40e-05) | (1.92e-05) | (1.47e-05) | (2.01e-05) | (2.03e-05) |
| Democracy Age Cubic | -1.10e-07** | 3.39e-08 | 1.31e-07* | -2.83e-08 | 9.96e-08 | 1.79e-07** |
|  | (5.08e-08) | (5.02e-08) | (7.21e-08) | (5.80e-08) | (8.00e-08) | (7.52e-08) |
| Military Regime | 0.0135 | -0.0176 | 0.0311 | -0.0211 | -0.0270 | -0.0282 |
|  | (0.0273) | (0.0213) | (0.0312) | (0.0229) | (0.0344) | (0.0350) |
| Civil Liberties | 0.761*** | 0.857*** | -0.0323 | 1.192*** | 0.226*** | 0.0641 |
|  | (0.0789) | (0.0549) | (0.0598) | (0.0516) | (0.0731) | (0.0835) |
|  |  |  |  |  |  |  |
| Observations | 12,078 | 11,977 | 12,079 | 12,079 | 10,070 | 10,070 |
| R-squared | 0.620 | 0.665 | 0.680 | 0.632 | 0.465 | 0.376 |
| Number of Countries | 171 | 171 | 171 | 171 | 171 | 171 |
| Country FE? | YES | YES | YES | YES | YES | YES |
| Year FE? | YES | YES | YES | YES | YES | YES |

Robust standard errors in parentheses

*** p<0.01, ** p<0.05, * p<0.1

**Table A6. Country- and year-fixed-effects models democracy and inequality on coups (5-year LDV)**

|  | (1) | (2) | (3) | (4) | (5) | (6) |
| --- | --- | --- | --- | --- | --- | --- |
|  | Democracy in 5 yrs. | Electoral Democracy | Suffrage | Legal Equality | Egalitarian Component | Equal Distribution |
|  |  | Index |  |  | Index |  |
|  |  |  |  |  |  |  |
| Recent Neighbor’s Coup | 0.0203 | -0.000997 | -0.00981 | -0.0199* | 0.00727 | 0.000539 |
|  | (0.0160) | (0.0141) | (0.0118) | (0.0107) | (0.0129) | (0.0140) |
| Legislature Corruption | 0.0340** | 0.0358*** | 0.0220* | -0.0210* | 0.0381*** | 0.0336** |
|  | (0.0151) | (0.0120) | (0.0117) | (0.0109) | (0.0141) | (0.0158) |
| Military Dimension Index | -0.0547 | -0.135*** | 0.0286 | 0.0737* | -0.0196 | -0.0915 |
|  | (0.0491) | (0.0463) | (0.0446) | (0.0435) | (0.0583) | (0.0775) |
| GDP/Capita | 0.00473 | 0.0427* | -0.0468* | -0.0497** | -0.0460 | 0.00858 |
|  | (0.0281) | (0.0237) | (0.0276) | (0.0209) | (0.0351) | (0.0345) |
| Economic Growth | 0.000401 | -0.000415 | 0.000370 | 0.00133** | 0.000288 | -0.000235 |
|  | (0.000652) | (0.000653) | (0.000628) | (0.000671) | (0.000571) | (0.000653) |
| Population | 0.0418 | 0.0218 | 0.0265 | 0.106*** | 0.152*** | 0.0712 |
|  | (0.0379) | (0.0315) | (0.0298) | (0.0297) | (0.0399) | (0.0546) |
| Number of Shocks (Past 5 yrs.) | 0.00601 | 0.00729 | 0.00875 | -0.00132 | 0.00127 | -0.00642 |
|  | (0.0128) | (0.00761) | (0.00929) | (0.00863) | (0.0111) | (0.0109) |
| Regional Democracy | -0.0381 | 0.0773 | 0.0221 | 0.0518 | -0.0590 | -0.111 |
|  | (0.0711) | (0.0639) | (0.0706) | (0.0515) | (0.0787) | (0.0916) |
| Past Democratic Spells | -0.000887 | 0.0341 | 0.0201 | -0.0414 | 0.0286 | 0.0502 |
|  | (0.0290) | (0.0248) | (0.0229) | (0.0281) | (0.0287) | (0.0428) |
| Democracy Age | -0.00678*** | -0.00177 | -0.000974 | -0.00301** | -0.000165 | 0.00187 |
|  | (0.00150) | (0.00128) | (0.00148) | (0.00119) | (0.00147) | (0.00150) |
| Democracy Age Squared | 4.37e-05** | 3.24e-06 | -1.58e-05 | 2.46e-05 | -1.78e-05 | -4.27e-05** |
|  | (1.89e-05) | (1.64e-05) | (2.00e-05) | (1.64e-05) | (2.05e-05) | (2.05e-05) |
| Democracy Age Cubic | -1.51e-07** | -5.40e-09 | 1.04e-07 | -6.51e-08 | 9.00e-08 | 1.74e-07** |
|  | (7.03e-08) | (5.93e-08) | (7.64e-08) | (6.50e-08) | (8.03e-08) | (7.70e-08) |
| Military Regime | 0.0161 | 0.0292 | 0.0292 | -0.0125 | -0.0403 | -0.0390 |
|  | (0.0295) | (0.0212) | (0.0321) | (0.0244) | (0.0309) | (0.0291) |
| Civil Liberties | 0.586*** | 0.687*** | -0.0414 | 0.917*** | 0.159** | 0.0408 |
|  | (0.0815) | (0.0576) | (0.0575) | (0.0528) | (0.0688) | (0.0811) |
|  |  |  |  |  |  |  |
| Observations | 11,444 | 11,360 | 11,445 | 11,445 | 9,592 | 9,592 |
| R-squared | 0.531 | 0.588 | 0.687 | 0.472 | 0.446 | 0.366 |
| Number of Countries | 170 | 170 | 170 | 170 | 170 | 170 |
| Country FE? | YES | YES | YES | YES | YES | YES |
| Year FE? | YES | YES | YES | YES | YES | YES |

Robust standard errors in parentheses

*** p<0.01, ** p<0.05, * p<0.1
